# Characterization of an Inorganic Powder-Based Scintillation Detector Under a UHDR Electron Beam

**DOI:** 10.3390/s24248064

**Published:** 2024-12-18

**Authors:** Daline Tho, Sam Beddar

**Affiliations:** 1 Department of Radiation Physics, The University of Texas MD Anderson Cancer Center, Houston, TX 77030, USA; dtho@mdanderson.org; 2 Medical Physics Program, The University of Texas MD Anderson Cancer Center UTHealth Houston Graduate School of Biomedical Sciences, Houston, TX 77030, USA

**Keywords:** FLAS, scintillator, scintillation, detector, inorganic, dosimetry

## Abstract

(1) Background: Ultra-high dose rate (UHDR) radiation therapy needs a reliable dosimetry solution and scintillation detectors are promising candidates. In this study, we characterized an inorganic powder-based scintillation detector under a 9 MeV UHDR electron beam. (2) Methods: A mixture of ZnS:Ag powder and optic glue was coupled to an 8 m Eska GH-4001-P polymethyl methacrylate (PMMA) optical fiber. We evaluated the dependence of the detector on dose per pulse (DPP), pulse repetition frequency (PRF), and pulse width (PW). Additionally, we determined the stability and the reproducibility of the detector. (3) Results: The signal ratio between the PMMA clear optical fiber and the ZnS:Ag scintillator was around 210. ZnS:Ag produced a signal yield 54 times greater than that of a BCF-12 plastic scintillator. Signal variation with PRF changes was under 0.5%. The signal was linear to the integrated dose up to the maximum deliverable dose, 180 Gy. The variation in signal was linear to the change in both PW and DPP. Regarding stability, the standard deviation of 10 consecutive irradiations was 0.83%. For the reproducibility, all daily measurements varied within ±1.5%. (4) Conclusions: These findings show that the ZnS:Ag detector can be used for accurate dosimetry with UHDR beams.

## 1. Introduction

Radiation therapy (RT) for cancer has become increasingly complex with modalities such as volumetric modulated arc therapy, image-guided therapy, and intraoperative RT. FLASH RT (FRT) delivers an ultra-high dose of radiation within a fraction of a second, as opposed to conventional treatment delivery, which takes minutes. Studies suggest that ultra-high dose rate (UHDR) RT more effectively spares healthy tissue compared to conventional treatment for equivalent administered doses [1,2,3]. Thus, FRT has the potential to significantly shorten treatment times and mitigate adverse effects related to toxicity in healthy tissues [1,2,4,5,6].

UHDR beams have many parameters, including dose per pulse (DPP), pulse repetition frequency (PRF), mean dose rate, and pulse width (PW), but the parameters involved in creating the healthy tissue-sparing effect remain unknown because of inconsistent dose reporting in different studies. A reliable detector would help researchers understand the FLASH effect by accurately measuring beam parameters as well as the dose delivered in real time.

Various detectors are under study for potential use in FRT dosimetry, such as beam current transformers (BCTs), ion chambers, and scintillator detectors [7,8,9,10]. The BCTs are capable of real-time measurements and can reliably monitor the beam at the exit of the accelerator. However, they do not allow for in vivo dosimetry, do not provide spatial information about the beam structure, and can only be used with charged particle beams [7]. Ion chambers are the standard dosimeters used in conventional RT, but they are subject to large changes in sensitivity because of ion recombination when used at UHDR [9,11,12]. In UHDR, the higher dose rate leads to higher ion density and increases the likelihood of recombination before the charges are collected by the electrodes. Finally, scintillator detectors are promising candidates for FLASH dosimetry due to their real-time measurement capability, dose linearity, and dose rate independence [13,14,15,16,17,18,19]. Another important property of a radiation detector dedicated to FRT is its radiation resistance and its behavior under a large, cumulated dose. Inorganic scintillators are more radiation resistant than organic scintillators [20].

In this study, we examined the response of an innovative inorganic ZnS:Ag powder scintillator detector under UHDR electron beams. The linearity of the dosimeter response with increasing dose, DPP, and mean instantaneous dose rate was validated. We also investigated the dependence of the ZnS:Ag scintillator on PW and PRF. Once the detector’s properties were determined, we determined its stability and reproducibility and compared the stem signal with that of an organic scintillator.

## 2. Materials and Methods

### 2.1. Detector Design

**Figure 1:**
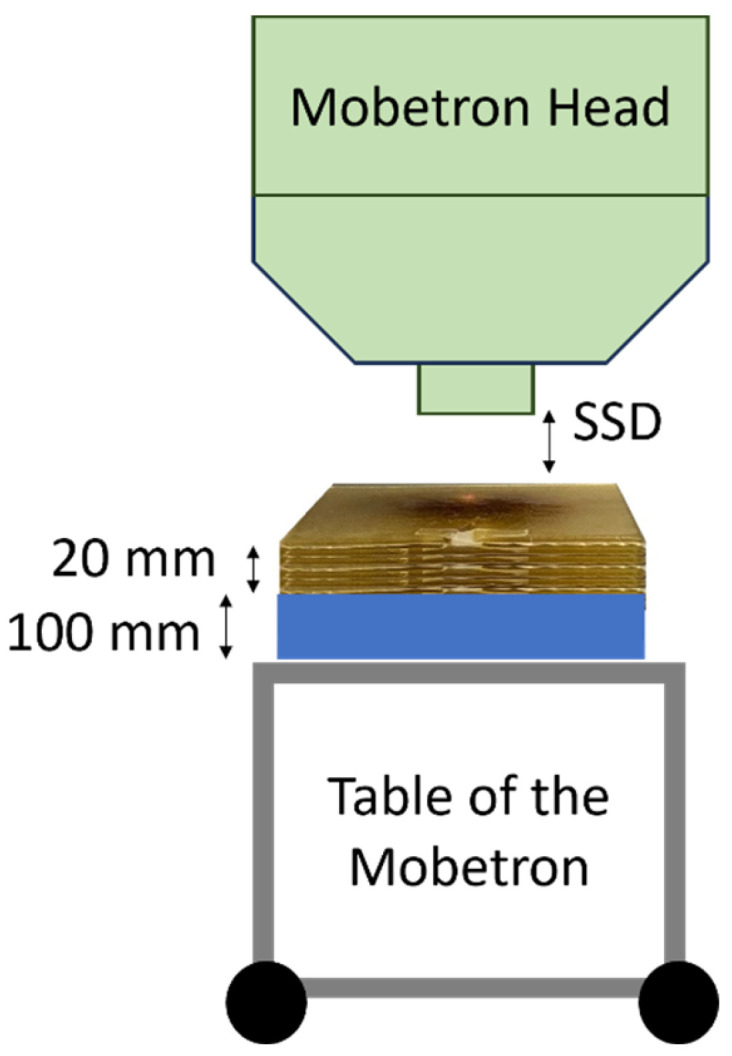
Setup for all measurements. The SSD corresponds to the distance between the Mobetron head exit and the surface of the setup.

To construct the detector, a ZnS:Ag powder (Eljen, Sweetwater, TX, USA) in a polyethylene terephthalate tube (Nordson Medical, Westlake, OH, USA) was coupled to an 8 m Eska GH-4001-P polymethyl methacrylate (PMMA) optical fiber (Mitsubishi Rayon Corporation, Tokyo, Japan) light guide. The powder typical particle size was 8 μm and it was mixed with Norland optical adhesive (Norland Products Inc., Jamesburg, NJ, USA) in a 1:100 mass ratio. The scintillator volume formed a cylinder measuring 0.2 mm in length and 1.0 mm in diameter. One end of the polished optical fiber was dipped into Norland optical adhesive and inserted into the tubing to connect with the scintillator volume. The other end of the optical fiber was connected to a Shamrock 163 spectrometer (Andor Technology, Belfast, Northern Ireland). The spectrometer was connected to a Lucas S charge-coupled device (CCD) (Andor Technology). For each acquisition, the whole field of the CCD camera was used, with an area of 6.58 mm × 4.96 mm. The camera’s elements were 10 × 10 μm^2^ each. The scintillator was placed at a 20 mm depth, with 100 mm of backscatter in all acquisitions (Figure 1). In this study, the surface-to-source distance (SSD) refers to the distance between the surface of the setup and the head of the irradiator. Electron beams of 9 MeV produced by a Mobetron FLASH electron linear accelerator (IntraOp, Sunnyvale, CA, USA) were used for all the irradiations [21]. For each spectrum acquired with the spectrometer, we determined the area under the curve (AUC), which represented the signal response of the detector. The parameters used for each experiment are presented in Table 1 [22].

### 2.2. Beam Parameter Variations

#### 2.2.1. Variation in PRF and Linearity of Dose

To investigate the mean dose rate dependence of the detector, we changed the PRF for 2 SSDs (Table 1). The mean dose rate tested ranged from 27 to 1008 Gy/s. The PW and the number of pulses were the same for both acquisitions. To assess the linearity of the signal to integrated dose, we incrementally increased the number of pulses delivered in a single acquisition. The number of pulses was between 1 and 120, corresponding to doses of 1.51 to 180 Gy.

#### 2.2.2. Variation in DPP

The DPP dependency of the detector was evaluated by changing PW but keeping the same SSD and PRF. The DPP value varied between 0.5 and 4.0 µs. The DPP dependency was also evaluated by varying the SSD from 0 to 150 mm at a fixed PW and PRF.

#### 2.2.3. PW and Instantaneous Dose Rate

To evaluate the relationship between the PW and the instantaneous dose rate, the DPP was matched to 0.5 Gy and 1 Gy at different PW by varying the SSD.

### 2.3. Scintillator Intensity and Stability Assessment

We aimed to characterize the signal intensity of the scintillator powder by comparing its scintillation spectrum to that of BCF-12, a well-known plastic scintillator. A BCF-12 scintillator with a diameter of 1 mm and a length of 2 mm was coupled to 8 m of clear PMMA fiber. The stem signal was measured by shifting the scintillator volume out of the field while exposing the same length of clear fiber. From the spectrum, we calculated the AUC ratios for both BCF-12 and ZnS:Ag.

For 10 consecutive days, the detector was irradiated daily, involving 5 acquisitions of 5 pulses at 9 MeV with a PW of 4 µs, SSD of 20 mm, and DPP of 5.6 Gy. The standard deviation of these daily measurements was calculated to assess the daily stability of the detector. Additionally, the stability was evaluated by computing the standard deviation of 10 consecutive measurements taken under the same experimental conditions. A rest period of 15 min was observed between each measurement to ensure accuracy and consistency. The BCTs measurements were used to validate the stability of the output of the linear accelerator.

## 3. Results

### 3.1. Beam Parameter Variation

#### 3.1.1. Variation in PRF and Linearity to Dose

**Figure 2:**
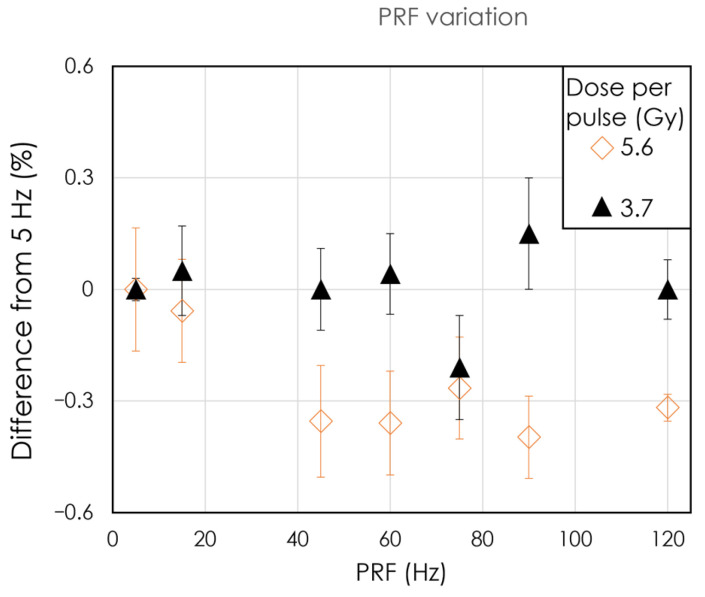
Detector signal normalized to the signal measured at 5 Hz as a function of pulse repetition frequency. Each data point is the average of triplicate measurements, and the error bar represents the standard deviation of the triplicates.

We first investigated the dependence of our detector’s signal on PRF. Figure 2 shows the difference in the percentage of the signal at the lowest PRF setting (5 Hz) as a function of PRF for DPPs of 5.6 Gy and 3.7 Gy. The absolute difference remained under 0.5% across all PRF values tested. At a DPP of 5.6 Gy, the detector signal was always lower than the reference at PRF = 5 Hz. The maximum absolute variation was 0.4% at 90 Hz, whereas the maximum absolute variation for DPP = 3.7 Gy was 0.2%. The mean dose rate tested ranged from 27 to 1008 Gy/s.

**Figure 3:**
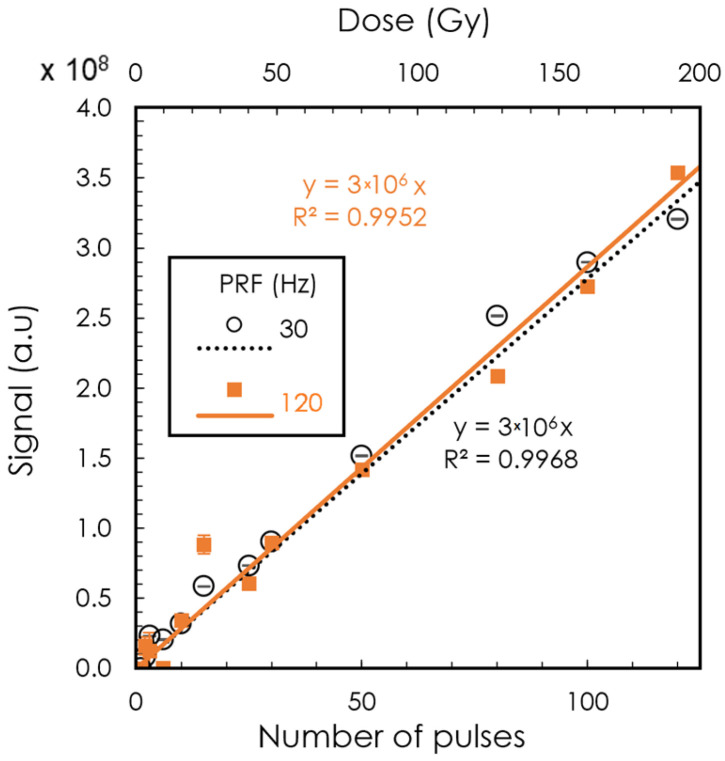
Signal as a function of dose. Variation in number of pulses at a DPP of 1.51 Gy for pulse repetition frequencies of 30 Hz and 120 Hz. The lower X-axis shows the number of pulses, and the top X-axis shows the corresponding dose.

After the PRF dependence study, we evaluated the detector signal as we increased the number of pulses delivered. Figure 3 shows the detector signal as a function of the number of pulses. The total delivered dose is indicated at the top of the graph. The signal was found to be linear to the integrated dose with an R^2^ greater than 0.99 for both PRF values tested (30 and 120 Hz).

#### 3.1.2. Variation in DPP

**Figure 4:**
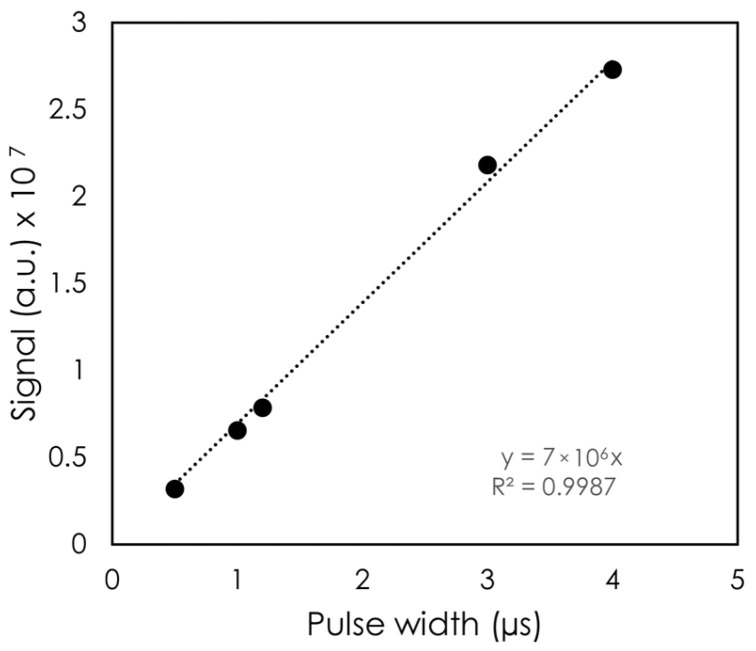
Dose per pulse response as a function of the pulse width. The error bars are too small to be visible.

The DPP was first varied by changing the PW. Figure 4 shows the signal as a function of PW. Across the PW values tested, the variation in signal was found to be linear with PW, with R^2^ > 0.99. The largest PW was 4 μs, which corresponded to a DPP of 4.5 Gy for this setup, and the shortest PW tested was 0.5 μs (DPP = 0.5 Gy).

**Figure 5:**
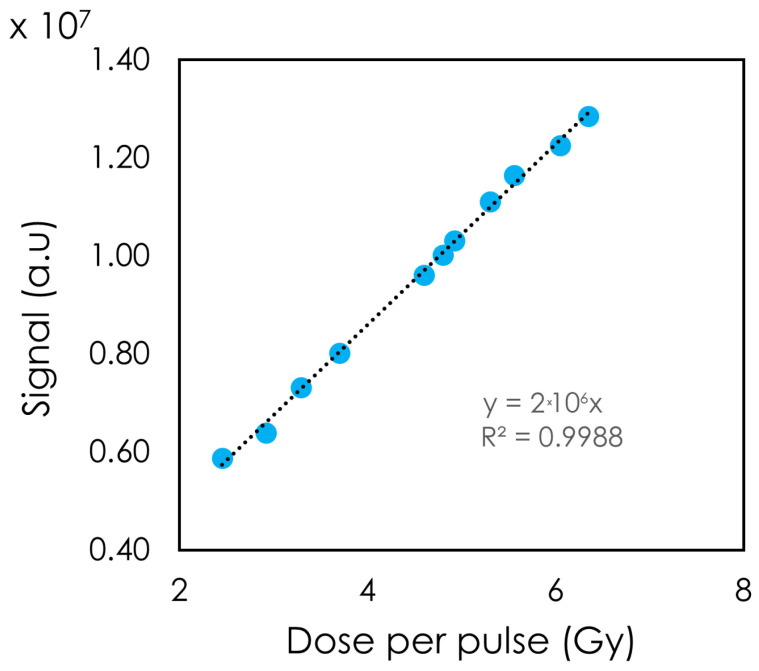
Signal of the detector for 1 pulse at different doses per pulse. The pulse width was 4 µs, and the pulse repetition frequency was 30 Hz.

The detector’s response was also tested by varying the DPP (Figure 5). A linear relation between the DPP and the detector’s light response was observed. DPPs of 2.45 to 6.35 Gy were achieved with a PW of 4 μs. At DPP = 2.45 Gy, the signal moved slightly away from the linear trendline.

#### 3.1.3. PW and Instantaneous Dose Rate

**Figure 6:**
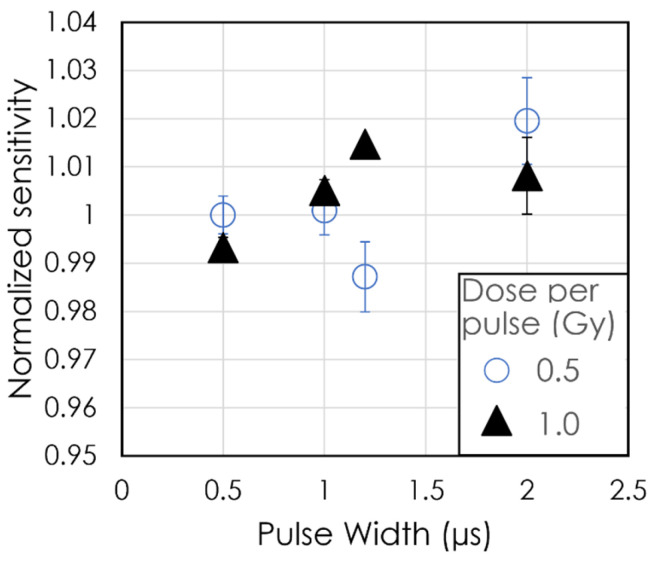
Normalized sensitivity as a function of pulse width. The data are normalized at a pulse width of 0.5 µs with a dose per pulse of 0.5 Gy. Each irradiation delivered 3 Gy to the detector. Each data point is the average of triplicate measurements, and the error bar represents the standard deviation of the triplicates.

In Figure 6, the dose sensitivity is displayed as a function of PW for matched DPPs of 0.5 and 1.0 Gy. All variations in sensitivity were within ±2% for both DPP values studied. The largest variation was 1.8% at PW = 2 μs for DPP = 0.5 Gy, while at DPP = 1.0 Gy, the maximum variation of 1.4% occurred at PW = 1.2 μs. The standard deviations at DPP = 1 Gy are smaller than at DPP = 0.5 Gy. As the PW increased, the normalized sensitivity of the detector at a DPP of 1 Gy was always higher than at PW = 0.5 μs.

### 3.2. Scintillator Intensity and Stability

**Figure 7:**
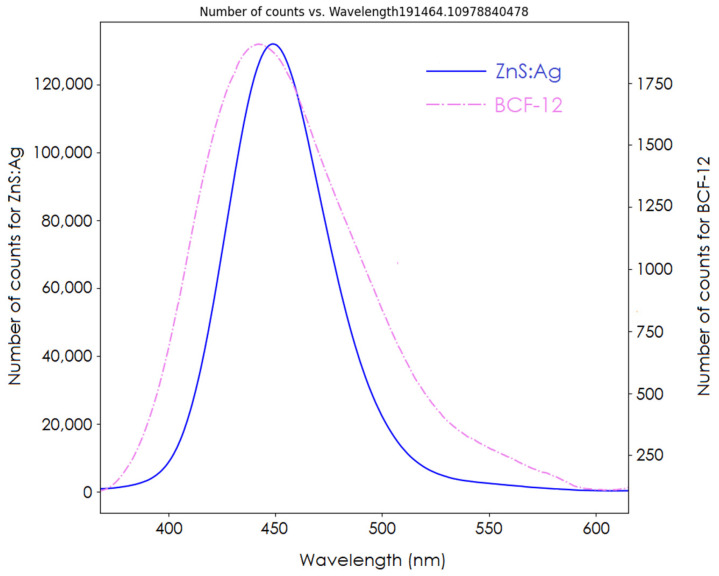
Spectra of ZnS:Ag and BCF-12.

The spectrum of our detector is shown in Figure 7. The signal ratios between the clear PMMA fiber signal and the BCF-12 and ZnS:Ag scintillators were 4.1 and 208.5, respectively. Comparison of the AUCs of both spectra showed that the inorganic ZnS:Ag scintillator had a signal yield 54 times that of the BCF-12 organic scintillator. From the acquired spectrum, the BCF-12 scintillator had an emission peak at 435 nm, shorter than that of the ZnS:Ag scintillator (450 nm).

**Figure 8:**
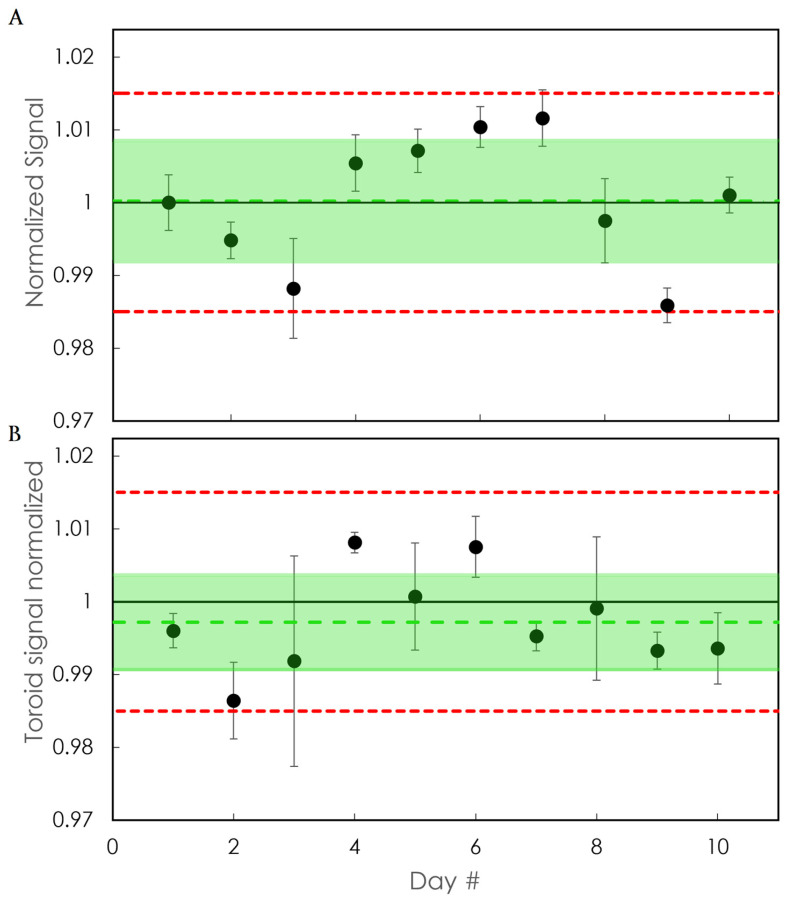
Response of (**A**) the detector and (**B**) the BCT to daily measurements for 10 days. The error bars correspond to the standard deviation of 5 consecutive measurements. The shaded area corresponds to 1 standard deviation from the mean. The dashed line in the middle of the shaded area is the mean normalized sensitivity. The dashed line in red corresponds to the ±1.5% deviation.

Regarding the reproducibility of the inorganic detector, Figure 8A displays the normalized sensitivity of the detector for all daily irradiations. The daily toroid signals are displayed in Figure 8B. All daily measurements were confined within ±1.5%. The highest deviations found were 1.2% for the detector’s normalized signal and −1.4% for the toroid signal.

**Figure 9:**
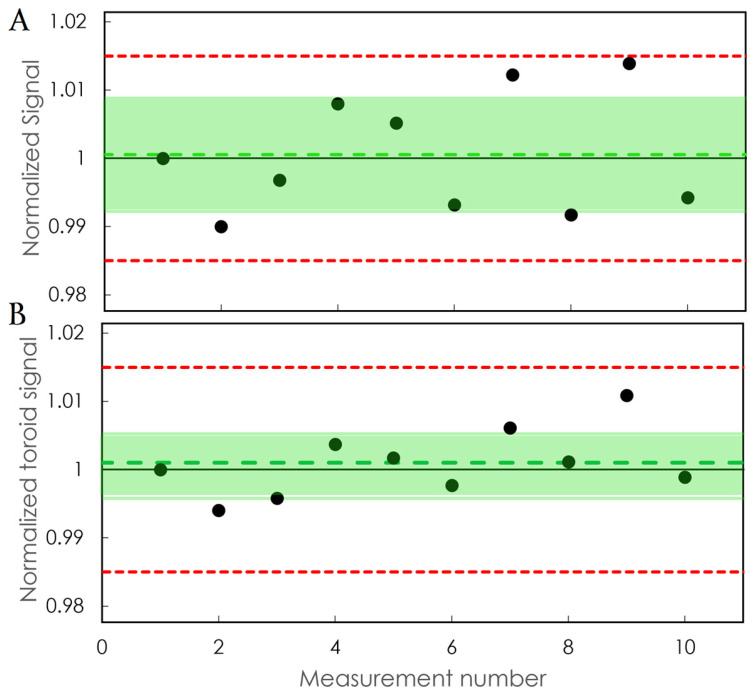
Response of (**A**) the detector and (**B**) the BCT to 10 consecutive measurements. Each measurement was followed by a 15 min rest period. The shaded area corresponds to 1 standard deviation from the mean. The green dashed line in the middle of the shaded area is the mean. The dashed line in red corresponds to the ±1.5% deviation.

Stability measurements are presented in Figure 9. Again, all the data points were within 1.5% of variation. We found stability of 0.83% for the inorganic detector. The standard deviation of the toroid signal was lower (0.47%) than the stability of the detector.

## 4. Discussion

We characterized an inorganic detector for FRT using ZnS:Ag powder as the scintillating volume. The data suggest that the ZnS:Ag detector was not dependent on the mean dose rate from 27 to 1008 Gy/s. The detector’s linearity response to the integrated dose was tested up to 180 Gy, the maximum dose that could be delivered to the detector in a single acquisition by our specific setup.

Varying the DPP via the PW or the SSD showed the detector’s linear response to dose. Figure 4 illustrates the linearity to PW, which in our setup translated to a higher DPP and a higher integrated dose. Changing the PW did not change the instantaneous dose rate, whereas changing the SSD did. The detector showed a linear response to DPP up to 6.35 Gy. At the lowest DPP (highest SSD), the signal deviated from the linear trendline. One possible explanation for this observation is that our fields were not collimated. As a result, with a high SSD, more clear fiber was exposed. Although we expected a greater stem signal with increasing SSD, no noticeable change in the inorganic scintillator’s spectrum was observed. This can be attributed to the high signal from the scintillator compared to the stem effect.

Other scintillation detectors show signal saturation; for instance, saturation starts at 1.5 Gy for the Exradin W2 (Standard Imaging) and at 3.5 Gy for cerium-doped lutetium (LYSO) [23,24]. Liu et al. (2024) suspected that the saturation originates from the photodetector rather than the scintillator itself. While the actual saturation point of the scintillator is unknown for the W2 system, correction for the stem effect is necessary with this scintillator. The reproducibility of the detector was found to be similar to the Medscint detector characterized by Baikalov et al. The advantage of using an inorganic scintillator is that it does not require this correction. A slower inorganic scintillator (Al_2_O_3_:C,Mg) has shown a decrease in light response with increasing PRF [25]. To avoid this PRF dependence, the scintillator needs to have a decay time shorter than the minimal inter-pulse time of the FLASH unit. In our case, the minimal inter-pulse time was 8 ms. The ZnS:Ag scintillator has a decay time of around 200 ns, which is low enough to avoid the decrease in light with increasing PRF. Di Martino et al. observed saturation with the clinical DoseVue 100 series point scintillator starting from 1 Gy/pulse [26].

When the dose was matched for each DPP and PW by changing the SSD, the sensitivity for DPP = 1.0 Gy was always higher than at PW = 0.5 μs. All sensitivity data were still within the uncertainty of 2%, which could be attributed to general variation in the linac output. We observed that the normalized sensitivity of the detector did not depend on the instantaneous dose rate between 250 and 2000 kGy/s. In addition, the stability and the reproducibility of our detector was comparable to the long-term and short-term stability of the same linac reported by Moeckli et al. during the commissioning process [27]. They found that the short-term stability for the 9 MeV electron beam of the Mobetron at commissioning was 0.8%, and the long-term stability over a period of 3 months was 1.7% for UHDR [25]. Beddar (2005) reported a standard deviation of 0.9% for a 9 MeV electron beam in conventional mode with the same unit after 20 daily quality assurance trials [28].

This study was limited by the beam parameters of the FLASH Mobetron. The limits in terms of PRF and PW were reached in this study. The beam’s energy dependency was not tested. Further tests with a wide range of beam energies will be needed for this detector because of the inorganic nature of ZnS:Ag.

## 5. Conclusions

In this study, we constructed a detector with an inorganic ZnS:Ag scintillator powder and characterized its dependence on different beam parameters. No dependence was found on PFR. The scintillator response was linear to the dose up to 180 Gy and to the DPP up to 6.36 Gy. This scintillator’s properties thus make it promising for the construction of detectors dedicated to UHDR beams. In addition, its powder state offers flexibility in terms of the desired shape of the detector. Future research should focus on validating the energy dependence and the radiation hardness of this scintillator.

## Figures and Tables

**Table 1:** Parameters of the beam for each experiment.

Experiment	PRF (Hz)	SSD (mm)	Pulse Width (μs)	Dose per Pulse (Gy)	# of Pulses
Variation in PRF	5–120	20	4.0	5.6	3
		80	4.0	3.7	3
Linearity to dose	30, 120	50	1.0	1.51	1–120
Variation in DPP via PW	30	50	0.5–4.0	0.5–4.5	1
Variation in DPP via SSD	30	0–150	4.0	2.45–6.35	1
PW dependence	30	0–200	0.5–2.0	0.5; 1.0	3; 6
Signal intensity to stem signal	30	20	4.0	5.6	1
Stability and reproducibility	30	20	4.0	5.6	5

DPP, dose per pulse; PRF, pulse repetition frequency; PW, pulse width; SSD, surface-to-source distance.

## Data Availability

Data are contained within the article.
